# Histone Deacetylase Inhibition and Dietary Short-Chain Fatty Acids

**DOI:** 10.5402/2011/869647

**Published:** 2011-12-26

**Authors:** Paul V. Licciardi, Katherine Ververis, Tom C. Karagiannis

**Affiliations:** ^1^Allergy and Immune Disorders, Murdoch Childrens Research Institute, Royal Children's Hospital, Flemington Road, Parkville, VIC 3052, Australia; ^2^Department of Paediatrics, The University of Melbourne, Parkville, VIC 3010, Australia; ^3^Epigenomic Medicine, Baker IDI Heart and Diabetes Institute, The Alfred Medical Research and Education Precinct, 75 Commercial Road, Melbourne, VIC 3004, Australia; ^4^Department of Pathology, The University of Melbourne, Parkville, VIC 3010, Australia

## Abstract

Changes in diet can also have dramatic effects on the composition of gut microbiota. Commensal bacteria of the gastrointestinal tract are critical regulators of health and disease by protecting against pathogen encounter whilst also maintaining immune tolerance to certain allergens. Moreover, consumption of fibre and vegetables typical of a non-Western diet generates substantial quantities of short-chain fatty acids (SCFAs) which have potent anti-inflammatory properties. Dietary interventions such as probiotic supplementation have been investigated for their pleiotropic effects on microbiota composition and immune function. Probiotics may restore intestinal dysbiosis and improve clinical disease through elevated SCFA levels in the intestine. Although the precise mechanisms by which such dietary factors mediate these effects, SCFA metabolites such as butyrate also function as histone deacetylase inhibitors (HDACi), that can act on the epigenome through chromatin remodeling changes. The aim of this review is to provide an overview of HDAC enzymes and to discuss the biological effects of HDACi. Further, we discuss the important relationship between diet and the balance between health and disease and how novel dietary interventions such as probiotics could be alternative approach for the prevention and/or treatment of chronic inflammatory disease through modulation of the intestinal microbiome.

## 1. Introduction

The histone deacetylase inhibitors (HDACi) suberoylanilide hydroxamic acid (SAHA, Vorinostat, Zolinza) and depsipeptide (Romidepsin, Istodax) have been approved by the US Food and Drug Administration (FDA) for the treatment of refractory cutaneous T-cell lymphoma, in 2006 and 2009, respectively [1, 2]. Further, at least 15 HDACi are currently undergoing clinical trials either alone or in combination with other therapeutic modalities for the treatment of various haematological and solid malignancies [3–5]. Apart from hydroxamic acids (e.g., SAHA) and cyclic peptides (e.g., depsipeptide), the short-chain fatty acids (SCFAs) represent another class of HDACi with clinical potential [6]. A notable example is valproic acid (VPA), a simple SCFA (eight-carbon) with more than 40 years clinical history in the treatment of epilepsy [7]. The mechanism of action of VPA in epilepsy is not fully understood, however, it is known to increase levels of the inhibitory neurotransmitter, *γ*-aminobutyric acid in the brain [8, 9]. In more recent years, VPA has also been shown to possess HDACi activity resulting in differentiation, cell death, and apoptosis in malignant cells [10–12]. Indeed, VPA is currently in phase II clinical trials for the treatment of glioblastoma multiforme in combination with radiotherapy and the chemotherapeutic temozolomide [13]. The aim of this review is to discuss the properties of dietary SCFA with an emphasis on probiotic metabolites such as butyrate.

## 2. Chromatin Modifications: Histone Acetylation

In eukaryotes, DNA is tightly packaged into nucleosomes, the fundamental subunits of chromatin [14]. Briefly, nucleosomes consist of approximately 146 base pairs of two loops of DNA wrapped around an octamer of two copies of each of the four core histones, H2A, H2B, H3, and H4 [14]. This organization is important for DNA metabolism, including transcription, replication, and repair. Chromatin conformation is dynamic and is strongly regulated by post-translational modifications of the histones which include acetylation of lysines, methylation of lysine and arginine residues, isomerization of proline, ADP-ribosylation, ubiquitination, and sumoylation [15].

The most well-characterized posttranslational histone modification affecting chromatin architecture is acetylation [15–17]. Histone acetylation status is regulated by the opposing actions of two groups of enzymes, namely, acetyl-transferases and HDAC. Acetyl-transferases transfer the acetyl group from acetyl coenzyme A to the *ε*-amino group of lysine residues [18]. This results in neutralization of the positive charge on histone tails and results in a more open, transcriptionally permissive chromatin conformation [18]. HDAC enzymes catalyze the removal of acetyl groups resulting in interaction of the negatively charged DNA with the positively charged histones [19]. This leads to a more compacted, transcriptionally repressive, chromatin conformation [16].

To date, 18 HDACs have been identified in mammals and these are divided into two distinct families. Firstly, the class III HDACs consist of sirtuins 1–7 which are homologous to the yeast enzyme silent information regulator (SIR) 2. These have an absolute requirement for NAD+ for removal of acetyl groups from acetyl-lysine residues [20–22]. The dietary antioxidant resveratrol is a well-known SIR1 agonist, having received much recent attention for its antiaging properties [23, 24]. The remaining 11 HDAC enzymes are known as the zinc-dependent HDACs and are typically referred to as the classical HDAC family [25, 26]. It is these enzymes that are inhibited by well-known broad-spectrum histone deacetylase inhibitors such as Trichostatin A, SAHA, depsipeptide, VPA, and sodium butyrate. The zinc-dependent HDACs are divided into three classes. Class I enzymes include HDAC1, 2, 3, and 8. These are related to the *Saccharomyces cerevisiae* transcriptional regulator RDP3 and in mammals they appear to have a major role in regulating cell survival and proliferation [27–29]. Class II is further subdivided into IIa consisting of HDAC4, 5, 7, and 9 and IIb comprising of HDAC6 and 10 which have two catalytic domains [30, 31]. Class II enzymes share homology with the yeast deacetylase HDA1 and are thought to have tissue-specific functions [4, 5, 32–34]. The sole member of class IV is HDAC11 which share similar features with both class I and II enzymes. Recent evidence indicates a role for HDAC11 in regulation of the immune system and in influencing immune activation versus immune tolerance [35, 36].

## 3. Biological Effects of Histone Deacetylase Inhibitors

Altered expression and aberrant recruitment of HDAC in malignancy provide the basis for the clinical potential of HDACi [4–6, 28, 33, 34, 37, 38]. HDACi are classified into different classes, the most potent being the cyclic peptides including the clinically approved depsipeptide as well as apicidin and trapoxin [1, 4, 5, 28]. The hydroxamic acids which include the prototype HDACi, Trichostatin A, and the clinically approved SAHA are also very potent inhibitors exhibiting efficacy in the nanomolar and low micromolar range, respectively [3–6, 28, 39]. The ketones such as *α*-ketomides and trifluoromethyl ketone represent another HDACi [3–6, 28, 39]. The SCFA which include drugs that have been used clinically for years in nononcological applications such as VPA, phenylbutyrate, and sodium butyrate are also effective inhibitors of HDAC enzymes, with activity in the millimolar range [3–6, 28, 39]. Although cell-free assays indicate that the HDACi described above exhibit some specificity towards different HDAC isoforms, they are generally known as broad-spectrum inhibitors as they inhibit enzymes in the different classes of zinc-dependent enzymes. For example, sodium butyrate inhibits most zinc-dependent HDACs except class IIa HDACs 6 and 10 [40].

**Figure 1:**
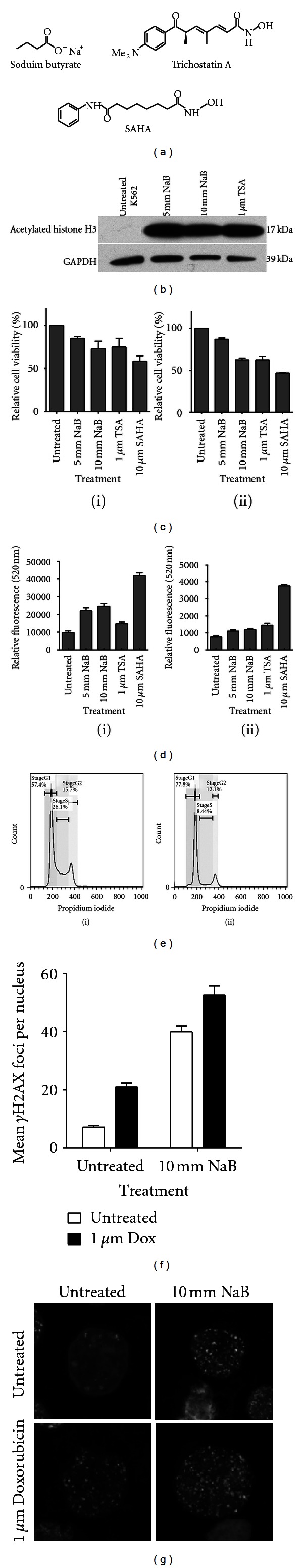
Snapshot of the effects of histone deacetylase inhibitors (HDACi) in human cells. (a) Chemical structures of sodium butyrate (NaB), Trichostatin A (TSA), and suberoylanilide hydroxamic acid (SAHA). (b) HDACi result in the hyperacetylation of histones. Immunoblots of acetylated histone H3 and loading control GAPDH in human peripheral blood monocyte cells treated with and without NaB and TSA at the indicated concentrations for 24 hours prior to whole cell lysate extraction. (c) HDACi decrease cell viability in (i) K562 cells and (ii) HT29 cells. Cells were treated with NaB, TSA, and SAHA with the indicated concentrations for 24 hours and relative cell viability was measured using the Cell Titre assay kit (Promega). (d) HDACi induce apoptosis (caspase 3/7 activity) in (i) K562 cells and (ii) HT29 cells. Cells were treated with and without HDACi for 24 hours and caspase 3/7 activity was measured using the Apo-one assay kit (Promega). (e) NaB alters the cell cycle distribution of K562 cells. Cells were either (i) untreated or treated with 10 mM NaB for 24 hours prior to staining with propidium iodide and analysis for cell cycle distribution using flow cytometry. (f) Sodium butyrate augments doxorubicin-induced DNA double-strand breaks (*γ*H2AX foci) in K562 cells. Cells were treated with and without 10 mM NaB for 24 hours prior to one-hour incubation with 1 *μ*M doxorubicin. Cells were washed and incubated in fresh media for a further 24 hours and stained for *γ*H2AX. Images were acquired using a Ziess 510 Meta confocal microscope and analyzed using ImageJ. (g) Photomicrographs indicating *γ*H2AX in K562 cells treated with doxorubicin or a combination of the anthracycline with NaB.

The biological effects of broad-spectrum HDACi in cancer cells are summarized in Figure 1. Treatment of malignant cells with HDACi results in the accumulation of hyperacetylated histones [5, 6, 27, 39]. Numerous nonhistone proteins such as *α*-tubulin, p53, Ku70, and heat shock protein 70 are also targets of HDACi [5, 6, 27, 39]. The effect of inhibition of HDAC enzymes is altered gene transcription; between 2–20% of genes are affected [5, 6, 27, 39]. Typically, HDACi induce cell death, altered cell cycle distribution, perturbed migration, and angiogenesis and activation of the extrinsic and/or intrinsic apoptotic pathways in malignant and transformed cell lines [4, 5, 28, 41]. Importantly, the effects of HDACi are much more profound (at least by a factor of 10) in cancer and transformed cells compared to normal cell lines providing a therapeutic window for clinical efficacy. Broad-spectrum HDACi have also been shown to exert additive and/or synergistic effects with other conventional cancer modalities such as chemotherapy and radiotherapy [24, 33, 42]. Interestingly, the use of HDACi combined with radiation dates back to the 1980s when sodium butyrate was found to potentiate ionizing radiation-induced cell death in cultured cells [43–45]. Further studies indicated that the SCFA sodium phenylbutyrate enhances radiosensitivity in nasopharyngeal carcinoma cells [46]. Indeed, it is widely accepted that HDACi will be most useful clinically when used in combination with other cancer modalities.

## 4. The Role of Diet in Health and Disease

In recent times, there has been considerable effort to delineate the important contribution of diet in health. While on the surface, this may seem an obvious link to draw, the development of cutting-edge microbiological technologies has made it possible to examine the precise mechanism by which diet influences health outcomes. Perhaps the most significant epidemiological evidence available is the increasing prevalence of diseases such as obesity (part of the metabolic syndrome), type 1 diabetes, and allergy attributed to the “Western” lifestyle [47]. Over the last 20 years, these problems are said to have reached epidemic proportions [48]. The relatively low incidence of these diseases in countries (e.g., Japan) where there are major differences in dietary consumption has also helped sharpen the focus on diet.

The relationship between gut microbiota and health has received a lot of attention in the past several years. The human intestinal microbiome contains up to 10^14^ bacterial species (mostly in the colon) that belong to approximately 1,000 different species [47]. However, only about 40 of these species constitute 99% of the total composition. Gut microbiota has an essential role in energy homeostasis, immune regulation, and protection against pathogens. The findings that diet can have profound effects on gut microbiota have given rise to the idea that dietary factors could be a major regulator of such epidemic diseases and other chronic inflammatory conditions. Data from animal experiments have demonstrated that germ-free mice had 40% less body fat than conventionally raised mice that could be reversed following colonization of their gut [49]. Furthermore, the microbial composition of mice given a “Western” diet containing high fat and sugar content could be modified after only 1 day, with lower numbers of beneficial *Bacteroidetes *species [50]. Germ-free mice also have an impaired ability to develop oral tolerance [51] to food or aeroallergens, demonstrating an important connection between a healthy microbiome and immune regulation.

## 5. Gut Microbiota, Short-Chain Fatty Acids, and Health

Modulation of the intestinal microbiome has wider impacts on immune regulation and inflammatory pathways. Indeed, several lines of evidence have shown that intestinal dysbiosis (imbalance of microbiota) is a feature of chronic disease as well as diet. Population differences in microbiota composition are well known; the profiles of gut bacteria in American, European, and Asian cohorts are quantifiably different [52–54]. Furthermore, gut microbiota is altered in allergic diseases and other chronic inflammatory disorders such as Crohn's disease [55]. For example, infants with eczema have reduced *Bifidobacteria* and elevated *Clostridia* and *Staphylococci *numbers in the intestine compared to healthy infants [56, 57] and these changes precede the development of allergic disease. In addition, reduced diversity in the faecal microbiota of infants with eczema at 1 week of age was observed compared to healthy infants [58]. In obese adults, lower levels of *Bacteroidetes* compared to lean controls have been reported [59] as well as markedly reduced bacterial diversity [60]. The fact that gut microbiota is modifiable by diet has provided a promising avenue for therapeutic targeting.

One of the major functions of gut microbiota is the digestion and fermentation of dietary fibre. This produces large quantities of SCFAs such as butyrate, acetate, and propionate, which have important roles in the regulation of immune and inflammatory responses. Under conditions of intestinal dysbiosis, the levels of SCFAs are markedly reduced as a result of the presence of aberrant microbial species. Butyrate, acetate, and propionate have all been found to be lower in allergic [61–63] or inflammatory bowel disease [64, 65] patients compared to healthy controls, suggesting that these could be markers of disease risk. The regulatory role of SCFAs has been further demonstrated in animals studies, where mice deficient in the GPR43 receptor (through which acetate signals) have increased inflammatory lesions in arthritis, allergic airways inflammation, and colitis models [66]. As GPR43 is expressed predominately on innate immune cells such as neutrophils, it is clear that deficient or altered SCFA levels as a function of diet or microbiota status can modulate disease susceptibility where the combination of these factors (as well as other host and environmental stresses) represent important risk factors of disease. The innate immune system is critical for sensing danger signals in the host as well as recognizing conserved pathogen-associated molecular patterns (PAMPs) during infection or in the case of gut microbiota, microbial-associated molecular patterns (MAMPs) via specific receptors such as TLRs or NLRs [67]. Mice deficient in TLR5 develop altered microbiota and subsequent metabolic syndrome [68]. Innate immune triggering during inflammation is a key feature in the pathogenesis of diet-microbiota associated diseases such as obesity. Activation of inflammasomes such as NLRP3 during this process involves caspase-1 activation, proinflammatory cytokines, as well as MCP-1 chemokine secretion [69]. Mice lacking NLRP3 were found to be resistant to the development of high-fat-diet-induced obesity suggesting this is an important regulator of this response [70].

## 6. Dietary Short-Chain Fatty Acids

The realization that diet is a critical regulator of health is not new. In fact, the eminent Russian scientist, Elia Metchnikoff, first reported the health prolonging properties of yoghurt and the bacterial cultures that were attributed to this effect [71]. However, the demonstration that gut microbiota is altered in certain disease states and that diet can modulate this has provided a renewed impetus for the identification of novel dietary therapeutics.

Probiotics have recently been the focus of scientific interest for their pleiotropic effects on host responses. Probiotics are defined as live microorganisms, which when administered in adequate amounts, confer a health benefit on the host [72]. The biological properties of probiotics are diverse and their effects are both species- and strain-specific and the two most well-studied species to date are *Lactobacillus *and *Bifidobacteria. *Interestingly, the characteristics of probiotics that make them well suited to the prevention and treatment of chronic inflammatory diseases include their ability to restore intestinal dysbiosis, maintain epithelial barrier integrity, and modulate various immune parameters [72, 73]. Prenatal treatment with the probiotic *Lactobacillus rhamnosus *GG (LGG) was shown to reduce the development of eczema in high-risk infants by 2 years of age [74]. Similar effects have also been demonstrated in Crohn's disease patients. In addition, probiotic treatment has also been associated with microbiota changes as well as regulation of immunity. Treatment with the probiotic *Faecalibacerium prausnitzii, *in a mouse model of colitis, was shown to correct the dysbiosis and reduce disease severity [75]. In clinical allergy trials, certain probiotics have been reported to modulate DC populations, Treg expression, and regulatory cytokine levels such as IL-10 and TGF-*β* [51, 76–79]. Immunomodulatory probiotics have also been shown to enhance oral vaccine-specific responses following polio [80], rotavirus [81], and typhoid [82] immunisation.

It has been recently proposed that probiotic dietary interventions mediate these effects in part through the restoration of microbiota and ultimately, SCFA levels. As discussed above, SCFA are critical regulators of microbiota and immune function and are also potent epigenome modifiers that exhibit histone HDAC inhibition activity [83]. Until now, this has not been clearly defined, however, preliminary evidence is emerging. In a recent study, prenatal administration of mice with *Acinetobacter lwoffii *F78 prevented asthma in the offspring in an IFN*γ*-dependent manner and was associated with histone H4 acetylation at the *IFNG* promoter [84]. Although SCFA levels were not directly assessed, it could be postulated that microbes (and probiotics) elicit epigenetic effects through critical anti-inflammatory and/or immunomodulatory molecules.

## 7. Conclusions

The role of diet has become increasingly important in the context of chronic inflammatory diseases such as allergy, inflammatory bowel disease, and cancer. Dietary manipulation of gut microbiota offers a promising approach for the prevention or treatment of these life-threatening conditions. Probiotics and their SCFA metabolites are a novel class of dietary substances that have the capacity to restore intestinal dysbiosis and regulate immune response and the combined effects on the microbiome, metabolome, and epigenome should be the subject of continued rigorous scientific examination.
